# A New Diagnostic Approach Using the NLR/LMR Ratio in the Differential Diagnosis of Neck Masses: Hematologic Evaluation of Patients With Cervical Lymphadenopathy

**DOI:** 10.1002/jcla.70297

**Published:** 2026-06-29

**Authors:** Servet Erdemes, Gizem Ay Haldız, Müslüm Ayral, Ali Çetin

**Affiliations:** ^1^ Department of Otorhinolaryngology Harran University Faculty of Medicine Şanlıurfa Türkiye; ^2^ Department of Pathology Harran University Faculty of Medicine Şanlıurfa Türkiye; ^3^ Department of Otorhinolaryngology Gazi Yaşargil Training and Research Hospital Diyarbakır Türkiye

**Keywords:** cervical lymphadenopathy, hematologic ratios, LMR, lymphoma, NLR, NLR/LMR, ROC analysis

## Abstract

**Objective:**

To evaluate the diagnostic utility of hematologic ratios derived from routine complete blood count parameters in differentiating malignant, granulomatous, and reactive cervical lymphadenopathies, with a particular focus on the novel neutrophil‐to‐lymphocyte ratio to lymphocyte‐to‐monocyte ratio (NLR/LMR).

**Methods:**

This retrospective study included 406 adult patients who underwent excisional cervical lymph node biopsy between 2015 and 2024 and had histopathologically confirmed diagnoses. Patients were classified into five groups: reactive lymphadenopathy, Hodgkin lymphoma (HL), non‐Hodgkin lymphoma (NHL), granulomatous non‐caseating (Gran–NC), and granulomatous caseating (Gran–C). Pre‐biopsy hematologic parameters (WBC, neutrophil, lymphocyte, monocyte, platelet, RDW) and derived ratios (NLR, LMR, PLR, RDW/PLT, NLR/LMR) were compared among groups. ROC curve analyses were used to assess the diagnostic performance of these parameters in distinguishing lymphoma from non‐lymphoma cases.

**Results:**

WBC, neutrophil, monocyte, and RDW levels were significantly higher in lymphoma groups than in reactive and granulomatous groups (*p* < 0.001). Among derived ratios, NLR, LMR, PLR, and NLR/LMR demonstrated significant intergroup differences (*p* < 0.001). ROC analysis identified NLR (AUC = 0.702, cut‐off = 2.02, OR = 4.92) and NLR/LMR (AUC = 0.707, cut‐off = 0.81, OR = 4.59) as the strongest discriminators for lymphoma. Multivariate logistic regression confirmed both NLR (OR = 1.49, 95% CI 1.24–1.79, *p* < 0.001) and NLR/LMR (OR = 1.94, 95% CI 1.46–2.57, *p* < 0.001) as independent predictors of malignancy.

**Conclusion:**

Simple hematologic ratios such as NLR and especially the combined index NLR/LMR can serve as reliable, low‐cost, and noninvasive diagnostic indicators for differentiating lymphoma from benign lymphadenopathies. Incorporating these ratios.

## Graphical abstract

This study evaluates complete blood count–derived inflammatory ratios for differentiating lymphoma from benign cervical lymphadenopathies. The novel NLR/LMR ratio demonstrated superior diagnostic performance (AUC = 0.71, OR = 4.6) compared with conventional indices. This simple and noninvasive biomarker may support clinical decision‐making and reduce unnecessary lymph node biopsies.

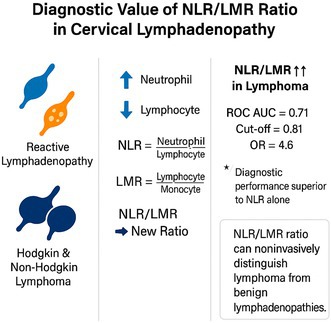

## 1 Introduction

Cervical lymphadenopathy (LAP) is a common clinical finding that may represent an early manifestation of various infectious, neoplastic, or inflammatory systemic diseases. Although the etiology of LAP is broad, distinguishing between benign and malignant causes remains a major diagnostic challenge. While reactive lymphadenitis is usually benign and self‐limiting, malignant conditions such as lymphoma can lead to serious consequences if not diagnosed promptly [1, 2].

Lymph node biopsy is considered the gold standard for definitive diagnosis; however, it is not feasible for all patients. The procedure carries inherent limitations due to its invasive nature, potential complications, and time‐consuming process [3]. Therefore, identifying noninvasive, rapid, and easily accessible parameters that can aid in the diagnostic process has become increasingly important.

In recent years, hematologic ratios derived from complete blood count (CBC) parameters—particularly the neutrophil‐to‐lymphocyte ratio (NLR), lymphocyte‐to‐monocyte ratio (LMR), and platelet‐to‐lymphocyte ratio (PLR)—have been proposed as diagnostic and prognostic markers in various malignant and inflammatory diseases, owing to their ability to reflect systemic inflammation [4, 5]. These ratios have also been reported to differ among malignant disorders such as lymphoma, granulomatous diseases like tuberculosis, and reactive conditions [6, 7].

Several studies have demonstrated significantly higher NLR and PLR values and lower LMR values in patients diagnosed with lymphoma [8]. Moreover, in both Hodgkin lymphoma (HL) and non‐Hodgkin lymphoma (NHL), these hematologic indices have been suggested to enhance diagnostic sensitivity and correlate with prognosis [4, 9]. In contrast, reactive lymphadenopathies generally exhibit normal or lower ratio levels [6].

Nevertheless, studies comparing these hematologic parameters across different lymph node pathologies remain limited. In particular, the diagnostic significance of newer indices such as the NLR/LMR and platelet‐to‐neutrophil (P/N) ratios has not been adequately clarified in the literature [10]. This study aims to evaluate the diagnostic utility of hematologic parameters in patients who underwent lymph node biopsy with histopathologically confirmed diagnoses and to identify the ratios that can distinguish lymphoma, granulomatous disease, and reactive lymphadenopathy.

## 2 Materials and Methods

This retrospective and descriptive study was conducted through a review of medical records of patients who presented with isolated cervical lymphadenopathy to the Otorhinolaryngology and Infectious Diseases outpatient clinics of Harran University Hospital between 2015 and 2024 and who underwent excisional lymph node biopsy. The study was approved by the Non‐Interventional Clinical Research Ethics Committee of Harran University Faculty of Medicine (Approval No: HRÜ/25.13.11). Only adult patients aged 18 years and older were included. Patients without systemic symptoms (such as fever, night sweats, or weight loss), with a histopathologically confirmed diagnosis, and with complete pre‐biopsy complete blood count (CBC) data were eligible for inclusion. Exclusion criteria were defined as immunodeficiency, active systemic disease, a history of hematologic malignancy, presence of non‐cervical lymphadenopathy, or incomplete laboratory records.

According to histopathologic diagnoses, patients were categorized into three main groups:

1. Reactive lymphadenitis,
2. Granulomatous lymphadenitis (secondary to causes such as tuberculosis or sarcoidosis), and
3. Lymphoma (including both Hodgkin and non‐Hodgkin subtypes).

For each patient, pre‐biopsy CBC parameters including white blood cell count (WBC), neutrophil, lymphocyte, monocyte, platelet, and red cell distribution width (RDW) were recorded. Derived hematologic ratios—neutrophil‐to‐lymphocyte ratio (NLR), lymphocyte‐to‐monocyte ratio (LMR), platelet‐to‐neutrophil ratio (P/N), and a novel composite index (NLR/LMR)—were calculated and analyzed. Complete blood count (CBC) analyses were performed using automated hematology analyzers (Sysmex series, Sysmex Corporation, Kobe, Japan) in accordance with routine clinical practice. Absolute neutrophil, lymphocyte, and monocyte counts were obtained directly from CBC results. The neutrophil‐to‐lymphocyte ratio (NLR) and lymphocyte‐to‐monocyte ratio (LMR) were calculated manually by dividing the respective absolute cell counts. The composite index NLR/LMR was subsequently derived from these two ratios.

### 2.1 Statistical Analysis

All statistical analyses were performed using IBM SPSS Statistics for Windows, Version 25.0 (IBM Corp., Armonk, NY, USA). The normality of numeric data was assessed using the Shapiro–Wilk test. For intergroup comparisons, the independent samples *t*‐test or one‐way ANOVA was used for normally distributed variables, whereas the Mann–Whitney *U* test or Kruskal–Wallis test was applied for non‐normally distributed data. Categorical variables were compared using the chi‐square test.

Receiver Operating Characteristic (ROC) curve analysis was performed to evaluate the diagnostic performance of hematologic parameters. The area under the curve (AUC), cut‐off values, and odds ratios (OR) were calculated for each parameter. A *p*‐value of < 0.05 was considered statistically significant.

## 3 Results

A total of 406 patients were included in the study. Of these, 86 (21.2%) had caseating granulomatous lymphadenitis, 70 (17.2%) had non‐caseating granulomatous lymphadenitis, 48 (11.8%) had Hodgkin lymphoma, 96 (23.6%) had non‐Hodgkin lymphoma, and 106 (26.1%) had reactive lymph nodes. When all cases were evaluated collectively, 168 patients (44.3%) were male and 238 (55.7%) were female. A statistically significant difference in sex distribution was found among the groups (*χ* ^2^ = 17.03, *p* = 0.0019). Post hoc analysis revealed that the higher proportion of females in the caseating granulomatous group and the higher proportion of males in the non‐Hodgkin lymphoma group accounted for this difference (*p* = 0.002).

**FIGURE 1:**
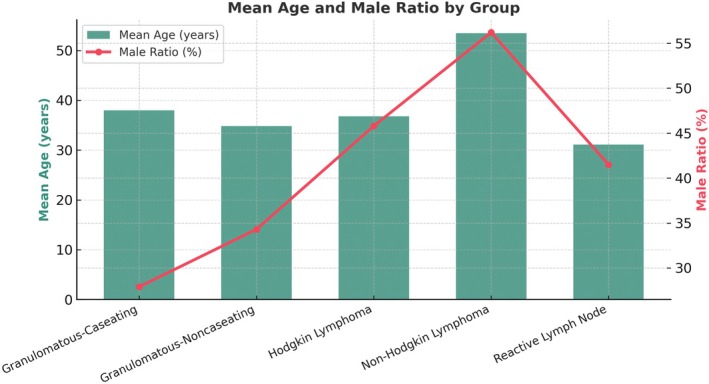
Age and sex distribution of patients according to diagnostic groups and subtypes.

The overall mean age of the patients was 39.3 ± 18.5 years, and a statistically significant age difference was observed among the groups (Kruskal–Wallis test, *H* = 67.14, *p* < 0.001). The reactive lymph node group represented the youngest cohort (31.1 ± 13.0 years), while the non‐Hodgkin lymphoma group had the oldest mean age (53.5 ± 21.0 years). Post hoc analysis demonstrated that patients with non‐Hodgkin lymphoma were significantly older than those in all other groups (*p* < 0.01), whereas patients with reactive lymph nodes were significantly younger (*p* < 0.01). The granulomatous and Hodgkin lymphoma groups had intermediate age distributions, with no statistically significant difference between them (Figure 1).

Table 1 presents the comparative distribution of hematologic parameters across the study groups. According to the Kruskal–Wallis analysis, statistically significant differences were observed in WBC, lymphocyte, monocyte, neutrophil, and RDW values (*p* < 0.05).

**TABLE 1:** Hematological parameters across diagnostic groups (mean ± SD).

Parameter	Reactive LN	HL	NHL	Gran–NC	Gran–C	*p*
WBC (×10^3^/μL)	8.18 ± 2.64	11.49 ± 6.22	10.64 ± 5.91	7.21 ± 2.18	8.24 ± 2.15	**< 0.001**
Lymphocyte (×10^3^/μL)	2.55 ± 0.79	2.16 ± 1.49	2.36 ± 1.33	2.28 ± 0.81	2.23 ± 0.67	**< 0.001**
Monocyte (×10^3^/μL)	0.57 ± 0.24	0.78 ± 0.37	0.77 ± 0.30	0.61 ± 0.26	0.69 ± 0.23	**< 0.001**
Neutrophil (×10^3^/μL)	4.79 ± 2.07	8.02 ± 5.57	6.10 ± 3.12	4.47 ± 2.08	5.03 ± 1.73	**< 0.001**
Platelet (×10^3^/μL)	291.78 ± 75.74	334.31 ± 119.83	305.76 ± 111.43	303.79 ± 97.52	288.17 ± 69.16	0.315
RDW (%)	12.49 ± 1.65	13.68 ± 2.85	13.03 ± 1.89	12.62 ± 1.67	12.91 ± 2.45	**0.016**

*Note:* Kruskal–Wallis test was used for intergroup comparisons. Bold *p*‐values indicate statistically significant differences (*p* < 0.05).

Abbreviations: Gran–C, Granulomatous lymphadenitis, caseating; Gran–NC, Granulomatous lymphadenitis, non‐caseating; HL, Hodgkin lymphoma; NHL, Non‐Hodgkin lymphoma; Reactive LN, Reactive lymphadenopathy.

WBC, monocyte, and neutrophil levels were markedly higher in both Hodgkin and non‐Hodgkin lymphoma groups compared to the reactive and granulomatous groups. Conversely, lymphocyte levels were elevated in the reactive group but reduced in the Hodgkin lymphoma group, which may reflect the hematologic manifestation of immune response activation and lymphoid tissue dysfunction.

The RDW values were significantly higher in the lymphoma groups, suggesting increased heterogeneity in erythrocyte production and potential bone marrow involvement. No significant difference was observed in platelet (PLT) counts among the groups (*p* = 0.315).

In addition, hematologic parameters in the granulomatous groups were generally similar to those in the reactive group but lower than in the lymphoma groups; however, these differences did not reach statistical significance.

Table 2 summarizes the distribution of hematologic ratios among the study groups. According to the Kruskal–Wallis analysis, statistically significant differences were found in NLR, LMR, PLR, and NLR/LMR ratios across groups (*p* < 0.001).

**TABLE 2:** Hematologic ratios across diagnostic groups (median [IQR]).

Parameter	Reactive LN	HL	NHL	Gran–NC	Gran–C	*p*
NLR	1.85 [1.40–2.09]	3.63 [2.55–4.82]	2.55 [1.94–3.54]	1.96 [1.41–2.48]	2.01 [1.64–2.80]	**< 0.001**
LMR	4.73 [3.24–5.89]	3.01 [1.68–3.57]	3.16 [2.04–4.51]	4.10 [2.98–5.12]	3.33 [2.60–4.10]	**< 0.001**
PLR	115.54 [88.77–156.54]	165.26 [120.40–247.73]	134.47 [102.29–201.42]	121.21 [98.05–190.68]	131.80 [97.13–170.17]	**< 0.001**
RDW/PLT (×10^3^)	43.56 [36.71–50.36]	40.60 [36.11–47.87]	40.37 [34.43–59.02]	41.39 [35.83–52.94]	45.08 [37.10–53.16]	0.591
NLR/LMR	0.38 [0.25–0.67]	1.28 [0.69–2.61]	0.84 [0.45–1.44]	0.48 [0.35–0.88]	0.62 [0.42–1.00]	**< 0.001**

*Note:* Bold values indicate statistically significant results (*p* < 0.05).

NLR and PLR values were significantly higher in the Hodgkin and non‐Hodgkin lymphoma groups compared with the reactive and granulomatous groups. This finding indicates the presence of marked neutrophilia and/or relative lymphopenia in lymphoma cases.

In contrast, the LMR ratio was higher in the reactive and granulomatous groups, suggesting that benign processes are characterized by a relatively preserved lymphocyte proportion and a lower monocyte response. Similarly, the NLR/LMR ratio showed a marked increase in the lymphoma groups, implying that this combined index may provide additional diagnostic value in differentiating malignant from benign lymphadenopathies.

No significant intergroup difference was observed in the RDW/PLT ratio (*p* = 0.591).

Table 3 presents the ROC analysis results of hematologic ratios used to differentiate lymphoma (HL + NHL) cases from other groups.

**TABLE 3:** Diagnostic performance of hematologic ratios for distinguishing lymphoma from non‐lymphoma groups.

Parameter	*p*	AUC	Cut‐off	OR
NLR	< 0.001	0.702	2.02	4.92
LMR	< 0.001	0.345	27.94	∞
PLR	< 0.001	0.613	179.80	3.01
RDW/PLT	0.2732	0.467	60.27	2.05
NLR/LMR	< 0.001	0.707	0.81	4.59

*Note:* The NLR and NLR/LMR ratios demonstrated the highest diagnostic performance for lymphoma detection, with approximately a 4–5‐fold increased risk of lymphoma observed in patients with values above the respective cut‐off thresholds.

Abbreviations: AUC, area under the curve; OR, odds ratio; ROC, receiver operating characteristic.

According to the analysis, NLR, LMR, PLR, and NLR/LMR ratios were found to be statistically significant (*p* < 0.001).

**FIGURE 2:**
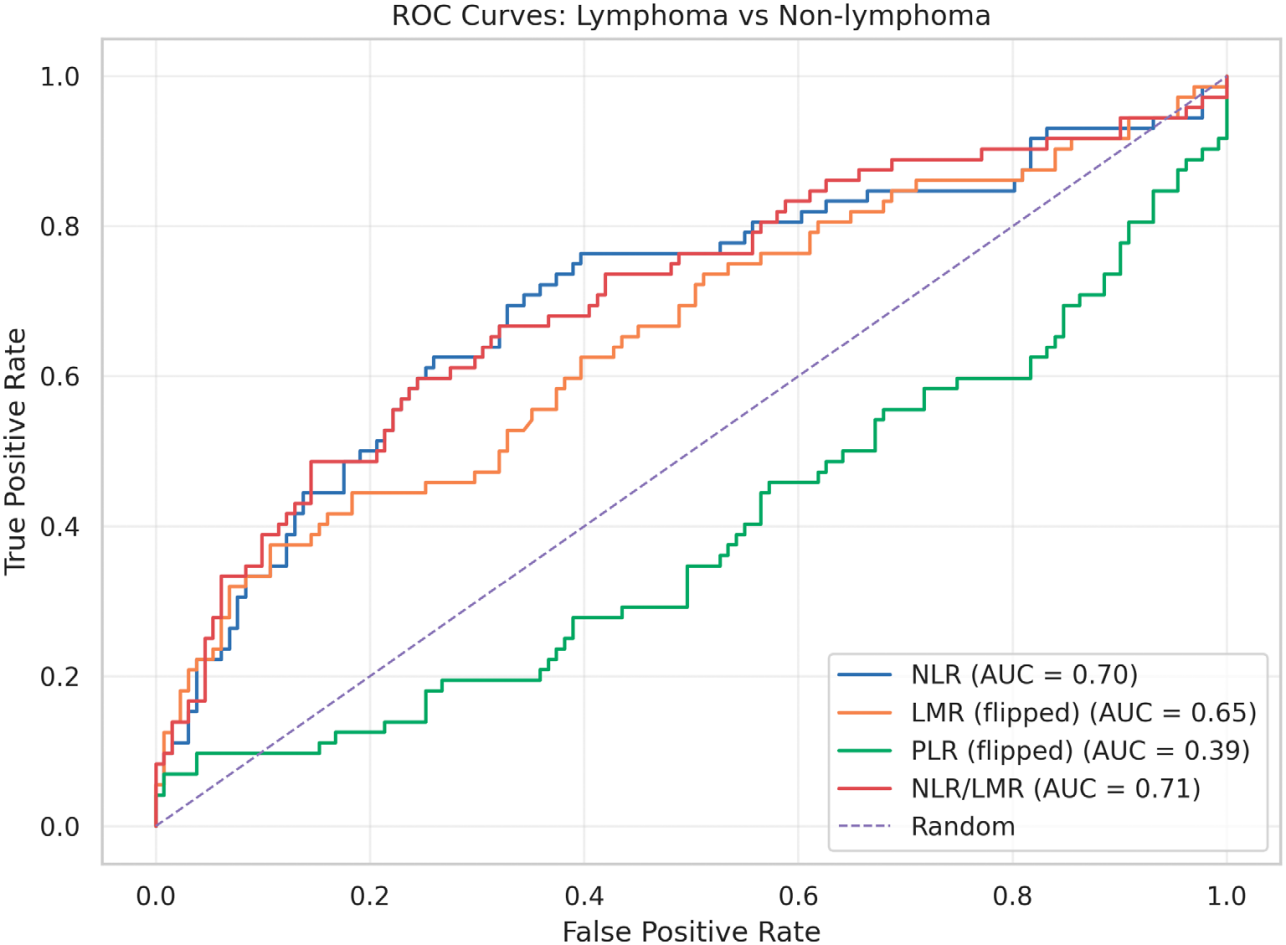
ROC curves for hematologic ratios distinguishing lymphoma from non‐lymphoma groups.

Among these parameters, the NLR and NLR/LMR ratios demonstrated the highest discriminative power for lymphoma diagnosis, with AUC values of 0.702 and 0.707, respectively (Figure 2).

Patients with values above the identified cut‐off thresholds (NLR ≥ 2.02 and NLR/LMR ≥ 0.81) had an approximately 4–5‐fold increased risk of lymphoma (OR = 4.9 and 4.6).

The PLR ratio also exhibited a moderate diagnostic ability (AUC = 0.613).

In contrast, the RDW/PLT ratio showed no statistically significant association with lymphoma (*p* = 0.27, AUC = 0.467).

These findings indicate that particularly NLR and NLR/LMR ratios, as simple hematologic indices derived from routine CBC parameters, may serve as reliable and clinically valuable diagnostic markers for differentiating lymphoma from benign lymphadenopathies.

Table 4 shows the results of the multivariate logistic regression analysis for hematologic ratios found to be significant in the diagnosis of lymphoma.

**TABLE 4:** Multivariate logistic regression analysis for predictors of lymphoma.

Variable	Model A OR (95% CI)	*p*	Model B OR (95% CI)	*p*
NLR	1.49 (1.24–1.79)	**< 0.001**	—	—
LMR	0.98 (0.89–1.09)	0.769	—	—
PLR	1.00 (1.00–1.00)	0.774	1.00 (1.00–1.00)	0.844
NLR/LMR	—	—	1.94 (1.46–2.57)	**< 0.001**

*Note:* Model A includes NLR, LMR, and PLR. Model B includes NLR/LMR and PLR to avoid multicollinearity. Lymphoma was coded as 1 (HL + NHL) and non‐lymphoma (Reactive LN + Gran–NC + Gran–C) as 0. Bold values indicate statistically significant results (*p* < 0.05).

*Model A* included NLR, LMR, and PLR as independent variables. The analysis revealed that only the NLR ratio remained an independent predictor of lymphoma (OR: 1.49, 95% CI: 1.24–1.79, *p* < 0.001). This finding indicates that each unit increase in NLR is associated with approximately a 1.5‐fold higher risk of lymphoma. Although LMR and PLR were significant in univariate analyses, they lost their independent predictive value in the multivariate model.

*Model B* included NLR/LMR and PLR variables, and the NLR/LMR ratio was identified as an independent and strong predictor for lymphoma (OR: 1.94, 95% CI: 1.46–2.57, *p* < 0.001). This composite index provides a more accurate prediction of lymphoma likelihood by combining the diagnostic contributions of NLR and LMR.

In both models, PLR was not statistically significant, and no multicollinearity issues were observed (VIF < 2).

These findings suggest that NLR and NLR/LMR can serve as independent hematologic indicators for lymphoma diagnosis, with the NLR/LMR combination offering enhanced diagnostic performance.

## 4 Discussion

Our study was designed to provide a new perspective on the use of hematologic ratios in the differential diagnosis of lymphadenopathy. One of the novel contributions of this research is the introduction of the NLR/LMR ratio, derived by combining two frequently used inflammatory indices, the neutrophil‐to‐lymphocyte ratio (NLR) and the lymphocyte‐to‐monocyte ratio (LMR) (AUC = 0.71). The comparative analysis of three distinct diagnostic categories—lymphoma, granulomatous lymphadenitis, and reactive lymphadenopathy—emphasizes the diagnostic value of assessing hematologic parameters not only individually but also as derived composite ratios. Furthermore, the inclusion of granulomatous lymphadenitis cases, which often represent a clinical “gray zone” in diagnostic practice, underscores the study's clinical relevance, extending beyond statistical significance to real‐world applicability.

Cervical lymphadenopathy is one of the most common clinical presentations encountered both in primary care and in specialized settings requiring advanced evaluation. Cervical lymph node enlargement may arise from various infectious, inflammatory, or neoplastic causes and requires careful assessment, particularly in adult patients due to the potential for underlying malignancy [1, 2]. Although physical examination, clinical history, and imaging studies play critical roles in differential diagnosis, histopathological examination remains the definitive diagnostic method in certain cases. Therefore, there is a growing need for noninvasive, easily accessible, and objective parameters that can aid in pre‐biopsy risk stratification.

Recent studies have highlighted the diagnostic and prognostic potential of complete blood count–based parameters, particularly derived indices calculated from neutrophil, lymphocyte, and monocyte series [4, 10]. These hematologic ratios have attracted increasing attention due to their simplicity, low cost, and ability to reflect systemic inflammatory status. Thus, CBC‐derived inflammatory indices are now considered valuable supportive tools in the diagnostic workflow for lymphadenopathy.

The hematologic manifestations of lymphadenopathy may vary not only by the underlying etiology but also with patient age. Previous studies have reported a physiological increase in neutrophil and monocyte counts and a relative decline in lymphocyte counts with advancing age [1, 2]. This tendency may contribute to the higher inflammatory index values observed in malignant conditions such as lymphoma, which are more common in older patients. Several investigations have also demonstrated that systemic inflammation in lymphoma is reflected in peripheral blood parameters, and these alterations may have diagnostic significance [8, 9, 11].

Atlıhan et al. reported significantly higher neutrophil and total WBC levels in patients with Hodgkin lymphoma compared with those with reactive lymphadenopathy [7]. Similarly, Dolu et al. found that monocyte counts were higher in malignant groups than in benign conditions [5].

Findings consistent with those reported in the literature were also observed in our study. In the Hodgkin lymphoma group, WBC, neutrophil, and monocyte levels were significantly higher than in both the reactive and granulomatous groups. The mean age of this group was also statistically higher. Taken together, these findings suggest that the inflammatory response is shaped not only by disease‐related factors but also by individual characteristics such as age, which may influence hematologic parameters. On the other hand, parameters such as platelet count and RDW did not differ significantly between groups. These basic hematologic trends provide a biological background that supports the clinical relevance of ratio‐based analyses.

Hematologic ratios have gained prominence in recent years as diagnostic and prognostic biomarkers for various solid and hematologic malignancies. Among these, the neutrophil‐to‐lymphocyte ratio (NLR) and lymphocyte‐to‐monocyte ratio (LMR) are recognized as reliable indicators that reflect the systemic inflammatory burden through peripheral blood counts. Numerous studies have demonstrated that these ratios provide valuable insights for both prognosis assessment and differentiation between benign and malignant diseases [4, 8, 9].

In recent years, the use of complete blood count–based inflammatory ratios has expanded beyond malignant diseases and has increasingly been explored in various infectious and inflammatory clinical settings. During the COVID‐19 pandemic, several studies reported that an increased neutrophil‐to‐lymphocyte ratio (NLR) and a decreased lymphocyte‐to‐monocyte ratio (LMR) were associated with a more severe clinical course and unfavorable outcomes. These alterations were interpreted as reflecting an imbalance between innate and adaptive immune responses in peripheral blood. In addition, it has been suggested that evaluating multiple hematologic parameters together, rather than relying on a single ratio, may provide additional value in clinical assessment. From this perspective, the present study examines the potential role of inflammatory ratios in the differential diagnosis of cervical lymphadenopathy and suggests that the NLR/LMR ratio may serve as a supportive parameter in distinguishing malignant from benign lymph node pathologies [12, 13].

In a study on patients with diffuse large B‐cell lymphoma (DLBCL), a pre‐treatment NLR cut‐off value of 3.0 was shown to significantly identify high‐risk groups, with individuals below this threshold exhibiting markedly better survival rates [14]. Similarly, Li et al. reported an LMR cut‐off of ≤ 2.6 with 72% sensitivity and 68% specificity (AUC = 0.78), and demonstrated that low LMR was significantly associated with poor prognosis [15, 16]. These results suggest that hematologic ratios should be considered not only as prognostic indicators but also as diagnostic tools [17].

In our study, both NLR and LMR demonstrated significant diagnostic differences and meaningful discriminative power in ROC analyses. These findings indicate that these ratios may serve as useful adjunctive parameters in distinguishing lymphoma from reactive and granulomatous lymphadenopathies. However, the most remarkable finding of our study was the high diagnostic performance of the derived composite parameter NLR/LMR, which combines these two indices. The AUC for this ratio was 0.71, with a cut‐off value of 0.81 yielding a sensitivity of 76% and a specificity of 71%. Moreover, patients with values above this threshold had approximately a 4.6‐fold higher likelihood of lymphoma compared to other groups (OR: 4.59). This parameter thus represents not only a statistically significant discriminator but also a potential clinically practical marker that may guide physicians in determining the need for biopsy [18].

In the existing literature, the NLR/LMR ratio has primarily been evaluated in gastrointestinal, pulmonary, and gynecologic malignancies, while its diagnostic role in hematologic cancers has rarely been investigated [10, 19]. Therefore, this study provides an original methodological contribution by demonstrating the potential utility of the NLR/LMR ratio in differentiating malignant from benign cervical lymphadenopathies, offering a new perspective for hematologic diagnostic algorithms. Given their low cost, noninvasive nature, and wide accessibility, such ratios may help reduce unnecessary biopsies and optimize clinical decision‐making, particularly in diagnostically ambiguous cases.

Granulomatous lymphadenitis represents a chronic inflammatory condition caused by etiologies such as tuberculosis or sarcoidosis, which may limit the diagnostic power of hematologic parameters. Although some studies have reported variations in hematologic ratios such as NLR and PLR in granulomatous diseases, their diagnostic performance remains generally limited. Kerget et al. found that both NLR and PLR levels were higher in tuberculosis and sarcoidosis cases than in reactive lymphadenopathy; however, their ROC analyses revealed low discriminatory power, particularly for differentiating tuberculosis from sarcoidosis (AUC ≈ 0.79 for PLR; AUC ≈ 0.85 for NLR) [20]. Similarly, Man et al. compared granulomatous and non‐granulomatous lymphadenitis and observed significant differences in NLR and PLR ratios, yet emphasized that these differences did not reach clinically applicable cut‐off levels in ROC‐based analyses [21].

These findings from the literature are consistent with the results obtained in the corresponding subgroup of our study. In the granulomatous group, no statistically significant differences were found among hematologic parameters; therefore, ROC analysis was not performed, and advanced analytical outputs such as cut‐off, sensitivity, specificity, or odds ratio were not calculated. This decision was based on the methodological principle that only parameters with statistically significant differences should be included in ROC analysis. Moreover, granulomatous diseases are typically observed in younger individuals and exhibit a heterogeneous clinical spectrum, which may cause hematologic responses to remain within normal reference ranges—thereby reducing the diagnostic discriminative capacity of ROC curves. Consequently, reporting the data of the granulomatous group descriptively at the table level was adopted as a methodological choice to maintain scientific validity and integrity.

Reactive lymphadenopathy represents benign lymph node enlargement that may arise from a variety of causes, including viral or bacterial infections, autoimmune disorders, and systemic inflammatory responses. Due to its broad clinical spectrum, this group inherently demonstrates heterogeneity in diagnostic studies. Previous research has reported that hematologic parameters in the reactive lymph node group generally present at lower levels than those in malignant groups. For example, systemic inflammatory markers such as NLR and PLR have been found to be significantly lower in reactive cases, although these differences did not yield strong discriminative power in ROC analyses [5, 7]. Several studies have also noted that parameters such as WBC, neutrophil, and lymphocyte counts can vary widely within this group, depending on factors such as infection type, age, and immune response intensity [2].

A similar pattern was observed in our study. The reactive lymph node group demonstrated a wide variation and several outlier values in WBC counts, reflecting a biological diversity that may contribute to diagnostic uncertainty. Nonetheless, when compared to malignant groups, certain hematologic ratios exhibited significant differences. In particular, NLR, LMR, and NLR/LMR ratios were found to be lower in the reactive group and significantly different from lymphoma groups in ROC analyses [16]. However, these differences did not provide diagnostic value within the reactive group itself but rather served as reference indicators when comparing benign and malignant entities. This finding suggests that while the reactive group functions effectively as a control group in ROC analyses, it does not independently yield strong diagnostic markers.

## 5 Conclusion

In the differential diagnosis of cervical lymphadenopathy, hematologic ratios derived from routine complete blood count parameters—particularly NLR, LMR, and especially NLR/LMR—may aid in the evaluation of patients with suspected malignancy. In our study, significantly higher WBC, neutrophil, and monocyte levels, along with elevated NLR and LMR ratios in the Hodgkin and non‐Hodgkin lymphoma groups compared to reactive and granulomatous groups reflect the impact of systemic inflammation in malignant processes. The strong discriminative performance of the NLR/LMR ratio in ROC analysis (AUC = 0.71, OR = 4.59) supports its potential clinical applicability.

This study demonstrates that noninvasive, low‐cost, and easily accessible hematologic ratios can serve as valuable tools in the diagnostic process of patients with cervical lymphadenopathy. The NLR/LMR ratio, in particular, may function as a decision‐support parameter in borderline or clinically indeterminate cases where the need for biopsy is uncertain. These results indicate that hematologic biomarkers can be utilized not only for prognostic purposes but also as effective diagnostic indicators. In this regard, our study provides a novel contribution to the literature by demonstrating the potential of the NLR/LMR ratio as a diagnostic biomarker for lymphoma. Further large‐scale, multicenter studies are warranted to validate these findings and to facilitate the integration of these ratios into clinical diagnostic algorithms.

## 6 Limitations

This study has several limitations. First, as a retrospective study, data were obtained from existing patient records, which may have led to missing information and limited access to detailed demographic or clinical variables. Although the number of patients in the granulomatous lymphadenopathy group was sufficient, ROC analysis was not performed due to the absence of statistically significant differences among hematologic parameters. Given the retrospective design and the long study period (2015–2024), different analyzer models within the same manufacturer series may have been used over time; however, all measurements were performed using standardized laboratory protocols, and all ratios were derived from absolute cell counts, minimizing potential analytical variability. Additionally, the reactive lymph node group consisted of heterogeneous etiologies, including infections and autoimmune conditions, without detailed subclassification of underlying causes. This heterogeneity may have contributed to the variability observed in hematologic parameters. Moreover, the hematologic ratios analyzed in this study (NLR, LMR, NLR/LMR) were evaluated solely for diagnostic purposes, excluding their potential prognostic implications.

Finally, the cut‐off and odds ratio values presented were derived exclusively from the current sample. Therefore, external validation through larger, multicenter cohorts is necessary to confirm the generalizability of our results.

## Funding

The authors have nothing to report.

## Ethics Statement

This study was approved by the Ethics Committee of Harran University Faculty of Medicine (Approval No: HRÜ/25.13.11). The study was conducted in accordance with the principles of the Declaration of Helsinki.

## Consent

Because this study was designed retrospectively, individual informed consent was not required. All patient data were anonymized prior to analysis.

## Conflicts of Interest

The authors declare no conflicts of interest.

## Data Availability

The data that support the findings of this study are available on request from the corresponding author. The data are not publicly available due to privacy or ethical restrictions.
